# Exploring Word-Adjacency Networks with Multifractal Time Series Analysis Techniques

**DOI:** 10.3390/e27040356

**Published:** 2025-03-28

**Authors:** Jakub Dec, Michał Dolina, Stanisław Drożdż, Robert Kluszczyński, Jarosław Kwapień, Tomasz Stanisz

**Affiliations:** 1 Faculty of Computer Science and Telecommunications, Cracow University of Technology, 31-155 Kraków, Poland; 2 Complex Systems Theory Department, Institute of Nuclear Physics, Polish Academy of Sciences, 31-342 Kraków, Poland; 3 Faculty of Mathematics and Computer Science, Jagiellonian University, ul. Łojasiewicza 6, 30-348 Kraków, Poland

**Keywords:** quantitative linguistics, complex networks, vertex observables, clustring coefficient, time series, multiscale correlations

## Abstract

A novel method of exploring linguistic networks is introduced by mapping word-adjacency networks to time series and applying multifractal analysis techniques. This approach captures the complex structural patterns of language by encoding network properties—such as clustering coefficients and node degrees—into temporal sequences. Using Alice’s Adventures in Wonderland by Lewis Carroll as a case study, both traditional word-adjacency networks and extended versions that incorporate punctuation are examined. The results indicate that the time series derived from clustering coefficients, when following the natural reading order, exhibits multifractal characteristics, revealing inherent complexity in textual organization. Statistical validation confirms that observed multifractal properties arise from genuine correlations rather than from spurious effects. Extending this analysis by taking into account punctuation equally with words, however, changes the nature of the global scaling to a more convolved form that is not describable by a uniform multifractal. An analogous analysis based on the node degrees does not show such rich behaviors, however. These findings reveal a new perspective for quantitative linguistics and network science, providing a deeper understanding of the interplay between text structure and complex systems.

## 1. Introduction

The application of the concept of complex networks [1,2,3,4,5] to the study of the organization of written texts is a very natural direction of research [6,7,8,9,10,11]. Complex networks are mathematical models used to represent interactions within large systems and, when applied to written texts, they provide insights into linguistic patterns, semantics, and the underlying structure of language [7,8,9,12,13,14,15]. In the context of written texts, words, phrases, sentences, or even entire documents can be modeled as nodes of a network, with edges representing relationships between them. Word-adjacency networks, also called word co-occurrence networks or word proximity networks, are a specific type of complex network where nodes represent words in a text, and edges represent their adjacency within a given context. These networks capture the local and global structure of how words are used together in language, revealing linguistic patterns, semantics, and the syntactic properties of texts [6,16,17,18]. There is strong evidence that, in a linguistic context, such networks have small-world properties [19]. However, there are possible different network architectures that can generate such behavior.

Previous studies have highlighted a potential connection between complex networks and time series [20,21,22,23]. Specifically, research provides evidence that time series can be transformed into networks—within the visibility graph scheme [24,25], for instance—and analyzed using techniques developed for complex network analysis [26]. Conversely, complex networks can be mapped onto time series [27], allowing for an alternative analytical approach. This latter transformation can be achieved using random walk frameworks on graphs, where, at each time step, a vertex property is ‘emitted’ by the walker. As a result, the generated time series may capture the structural organization of the network based on the chosen vertex property [28,29]. Keeping thus in mind the interplay between network theory and time series analysis [30,31], a study of the linguistic properties of word-adjacency networks is carried out here by mapping such networks to time series and analyzing such series using the related methodology.

## 2. From Word-Adjacency Networks to Time Series

This methodology and approximation are illustrated with the example of a book Alice’s Adventures in Wonderland by Lewis Carroll that is widely known and often used in quantitative linguistic studies [32]. The networks to be studied are constructed from such a text sample after linking items of interest that are direct neighbors of each other at least once in a sample. Two cases of such items are considered here. The first one takes the traditional approach of considering all words as nodes in a network. The second one also includes all punctuation marks in addition to all words and is dictated by the fact that punctuation marks appear [33] to obey Zipf’s law [34,35] on an equal footing with words. This book contains 2627 different words and includes punctuation 2636 items in total.

**Figure 1:**
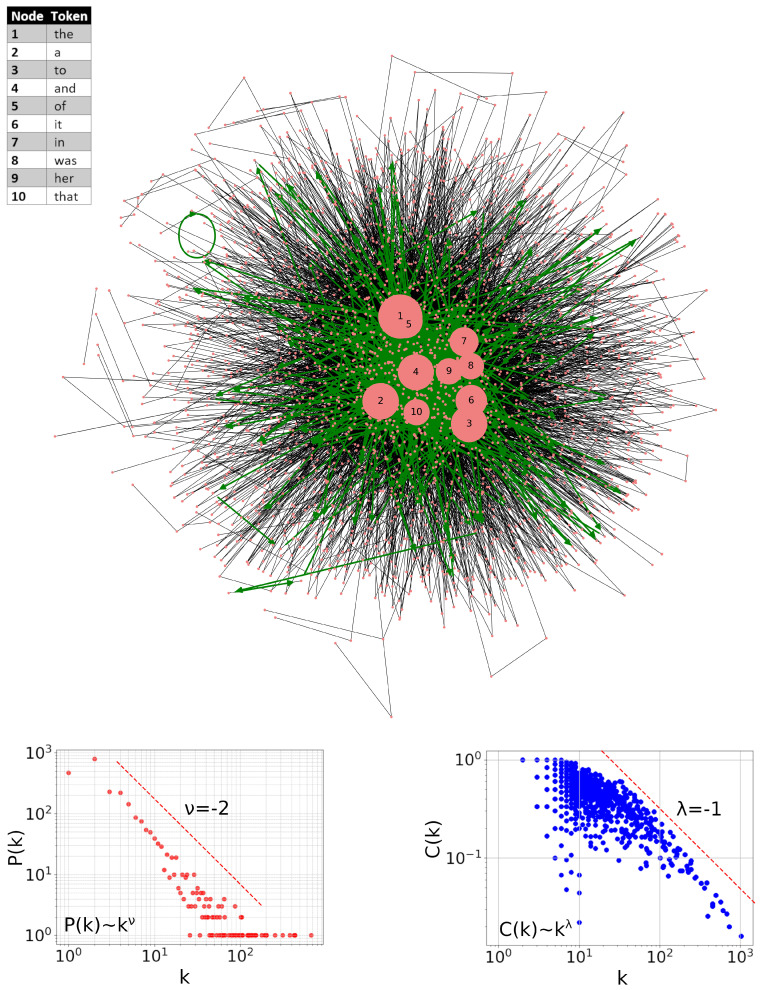
Visualization of the word-adjacency network of a book Alice’s Adventures in Wonderland by Lewis Carroll created exclusively from words. Top-10 hubs are pointed out explicitly. The green line indicates the path that corresponds to the actual reading order of this book. In order not to make this image too opaque, only the visit within the first 1000 words is marked here. Bottom-left inset displays the degree *k* distribution P(k) of this network, and the bottom-right one displays the corresponding distribution of the clustering coefficients C(k). The red dashed lines indicate the corresponding predictions for the model hierarchical networks.

The network created in this way for this book, taking into account words only, is shown in Figure 1. It appears to conform quite clearly to a hierarchical organization [1,2]. Top-10 hubs of this network are indicated explicitly. The resulting complete node-degree distribution P(k) follows the power-law trend indicated by the dashed straight line $k^{-2}$ (which corresponds to the Zipf’s law) in the bottom-left panel of this figure. Furthermore, as the bottom-right panel of this Figure shows, the correspondence between the distribution of clustering coefficients in the gross structure also follows the theoretical predictions $C\left(k\right)∼k^{-1}$ for hierarchical networks [36,37]. (For a node *i* with $k_{i}$ links (edges), these coefficients are defined in the usual way as $C_{i}=2n_{i}/k_{i}\left(k_{i}-1\right)$, where $n_{i}$ is the number of edges between the $k_{i}$ neighbors of *i*). Finally, the green line indicates the path across the first 1000 words that corresponds to the actual reading order of this book. As one can see, this line mainly travels around the area of greater concentration of hubs and occasionally moves away to the periphery of the network but keeps returning quickly. Interestingly, even the loop can be seen here (upper left part of the network). In this particular case, it reflects a sequence of the same words separated by commas. However, commas are not present in this network.

**Figure 2:**
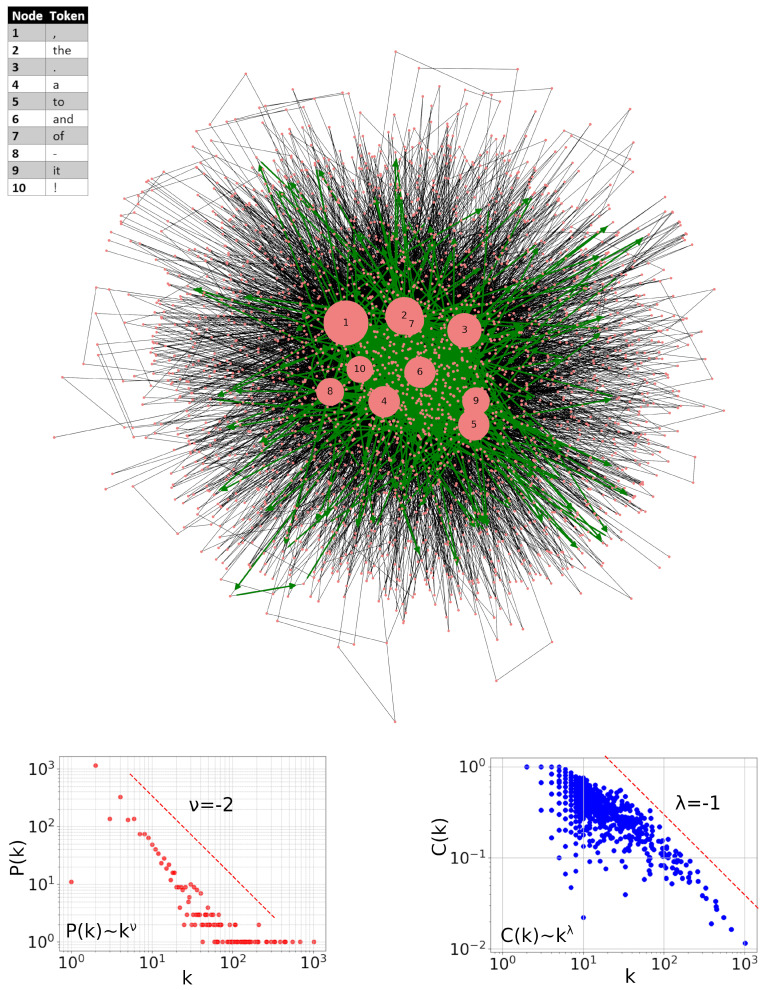
Visualization of the word-adjacency network of a book Alice’s Adventures in Wonderland by Lewis Carroll created from both words and punctuation marks. Top-10 hubs are pointed out explicitly. The green line indicates the path that corresponds to the actual reading order of this book. In order not to make this image too opaque, only the visit within the first 1000 items is marked here. Bottom-left inset displays the degree *k* distribution P(k) of this network, and the bottom-right one displays the corresponding distribution of the clustering coefficients C(k). The red dashed lines indicate the corresponding predictions for the model hierarchical networks.

In what follows, this network is therefore enriched by incorporating punctuation, and the resulting network is shown in Figure 2 with the same convention as before. Now, there are four punctuation marks among the top-10 hubs, which can significantly affect the dynamic characteristics of the reading trajectory shown, as before, in green. In particular, the loop commented above in Figure 1 has now disappeared. In the topological properties of the network taking into account punctuation marks, here expressed by the distributions of degree P(k) and local clustering coefficient C(k), the changes are not clearly significant.

A more systematic visit to them according to specific rules may provide deeper insight into the organization of such networks also in the sense of how they grow. Such rules are not rigidly defined for all tasks but will be dictated by the specificity of a given issue. In the current linguistic context, the natural order of node visits is in the direction of writing and then reading the text. A natural reference for this way of visiting the word-adjacency network is sampling it by a random walk. Of course, for a more complete, multi-dimensional view, various network characteristics can be used in both cases.

**Figure 3:**
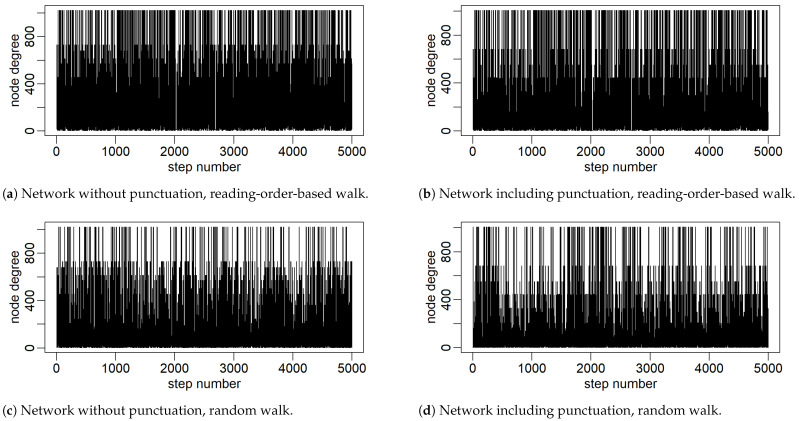
The first 5000 values of time series constructed from the degrees of nodes visited in a network walk. Two variants of the network are considered: without punctuation (**left column**) and with punctuation marks included in the node set (**right column**).

**Figure 4:**
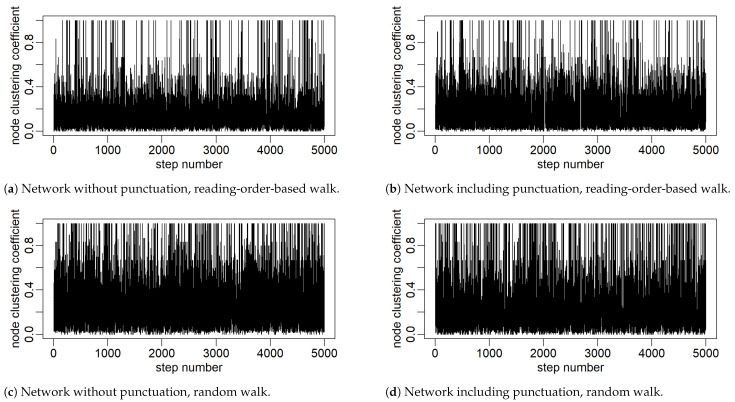
The first 5000 values of time series constructed from the clustering coefficients of nodes visited in a network walk. Two variants of the network are considered: without punctuation (**left column**) and with punctuation marks included in the node set (**right column**).

A few sample but representative cases are shown in what follows. Figure 3 shows the time series of node degrees *k* ‘emitted’ during visits along the word adjacency network and along the extended network, respectively, so that punctuation marks are also included on par with words. In both cases, a journey is conducted in accordance with the reading order and, independently, determined by the random walk rules. The resulting consecutive 5000 steps in each of the four cases are shown. An analogous composition of cases with the clustering coefficient ’emitted’ is shown in Figure 4. The obtained patterns of variability in these series show quite rich organization. A cursory visual inspection shows a slightly more varied organization of this variability when the clustering coefficient is used. In particular, for the case of a network without punctuation and generating a series in accordance with the reading order, it shows clustering of variability resembling the one known, for example, from the dynamics of financial markets, which typically implies self-similarity and fractals [38].

## 3. Detrended Fluctuation Analysis

Self-similarity, characterized by the absence of a distinct scale, is a fundamental trait of natural complex systems. Empirically, this property often appears as a distinct temporal structure in measurement outcomes, represented as time series data. In particular, complexity is commonly associated with a hierarchical cascade of data points exhibiting multiscaling, highlighting the need for effective methods to identify such patterns in complex systems research [39].

Extensive studies have documented that multifractal detrended fluctuation analysis (MFDFA) is a highly reliable technique for examining multiscaling patterns [40,41] and in the linguistic context [42,43,44,45]. Expanding on the widely utilized detrended fluctuation analysis (DFA) [46], MFDFA offers a multiscale framework for examining hierarchical and multiscaling behaviors. The key steps of the MFDFA algorithm are briefly outlined below.

### 3.1. Multifractal Formalism

A time series $U={\left\{u_{i}\right\}}_{i=1}^{T}$ of *T* consecutive measurements of an observable *u* is partitioned into $M_{s}$ non-overlapping windows of length *s*, starting from both ends of *U*. This results in a total of $2M_{s}$ windows. In order to properly handle potential non-stationarity in the signal, a detrending procedure is conducted within each window on an integrated signal, known as the profile, $X={\left\{x_{i}\right\}}_{i=1}^{s}$. Its elements are defined as:

(1) $$x_{i}=\sum_{j=1}^{i}u_{j}.$$

The detrending procedure involves fitting a properly matched low-order polynomial $P^{\left(m\right)}$ (with *m* typically between 1 and 3 depending on the degree of signal roughness) to the data *X* within each window $\nu =0,\ldots ,2M_{s}-1$ and then subtracting it. The variance of such a detrended signal is then determined as:

(2) $$f^{2}\left(\nu ,s\right)=\frac{1}{s}\sum_{i=1}^{s}{\left(x_{i}-P^{\left(m\right)}\left(i\right)\right)}^{2}.$$

Based on it, a family of fluctuation functions of order *r* is determined using the average variance computed across all windows:

(3) $$F_{r}\left(s\right)={\left\{\frac{1}{2M_{s}}\sum_{\nu =0}^{2M_{s}-1}{\left[f^{2}\left(\nu ,s\right)\right]}^{r/2}\right\}}^{1/r},$$

where *r* is a real number.

The fluctuation functions $F_{r}\left(s\right)$ are calculated for different values of the scale *s* and the index *r*. Typically, the minimum value of *s* is chosen to be greater than the longest sequence of constant values in *U*, while the maximum is set to a factor of a few smaller than *T*. In contrast to *s*, there is no universally defined range for *r*. Since *r* corresponds to the moments of the signal, extreme values should be avoided in time series with heavy tails to ensure meaningful results. The range of considered values of parameter *r* is limited by the possibility of the appearance of divergent moments in Equation (3). −10≤r≤10 is a fairly typical range used in the literature and is also appropriate in the present application. If the fluctuation functions follow power-law dependencies on *s*,

(4) $$F_{r}\left(s\right)∼s^{h(r)},$$

within such a range of *r* and, in addition, h(r) is *r*-dependent, this indicates that the time series under study is multifractal. Otherwise, when h(r) is constant in *r*, it is just monofractal. The function h(r) can be considered the generalized Hurst exponent, because h(r)=H for r=2, where *H* is the standard Hurst exponent [47,48]. Signatures of fractal organization of $F_{r}\left(s\right)$ result in straight lines on double logarithmic plots.

Characteristics of multifractality can thus be quantified directly in terms of h(r)-dependence, but another commonly adopted measure is the singularity spectrum f(α), which is derived from h(r) by the Legendre transform:

(5) $$\begin{array}{c} \alpha =h\left(r\right)+rh^{\prime }\left(r\right),\\ f\left(\alpha \right)=r\left[\alpha -h(r)\right]+1, \end{array}$$

Here, α reflects the data-point singularity, equivalent to the Hölder exponent. Geometrically, f(α) can be interpreted as the fractal dimension of the subset of the data characterized by a specific Hölder exponent α [49]. For a multifractal time series, f(α) typically forms a downward-pointing parabola. The broader the singularity spectrum f(α), the greater the richness of multifractality in the time series, which serves as an indicator of its complexity content. In some cases, the f(α) parabola may appear distorted or asymmetric, suggesting that data points of varying amplitudes exhibit different scaling behaviors [50,51,52,53,54,55]. For a monofractal time series, the pair (α,f(α)) converges to coordinates of a single point.

### 3.2. Fluctuation Functions and Their Scaling Characteristics

**Figure 5:**
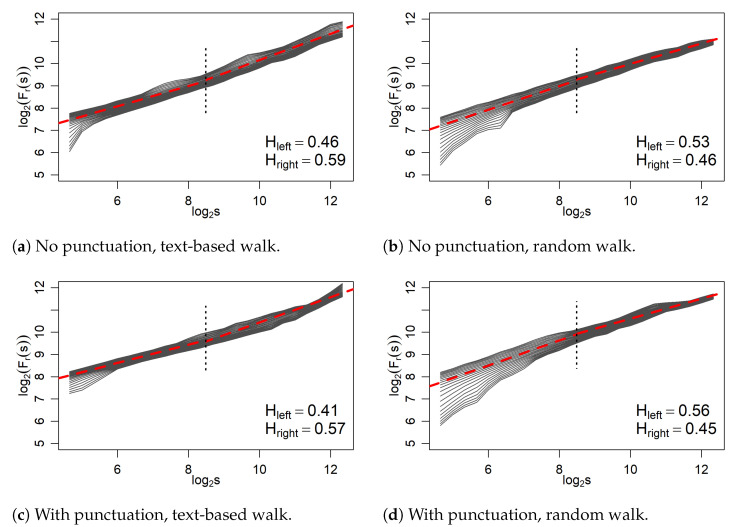
Fluctuation functions of time series constructed from the degrees of nodes visited in network walks. The linear fits to the points corresponding to r=2, separate for the left and the right halves of the plots, are marked by red dashed lines. The corresponding Hurst exponents are given in the bottom right corner of each plot.

All the most important characteristics of correlation in time series are contained in the fluctuation functions defined by the Equation (3); in their degree of scaling; and, above all, in their mutual relationship for different values of *r*. Four collections of fluctuation functions of time series constructed from the degrees of nodes visited during walks along networks of Figure 1 (words only) and Figure 2 (words and punctuation marks) are shown in Figure 5. The scaling effects are quite visible here, but this scaling is rather of the monofractal type because it is not diverse enough in *r* to indicate multifractality. If at all, more dependencies on *r* can be seen in the case of a random walk in this network, but downward deflections on small scales for negative values of *r* may result from the appearance of several consecutive similar values in a series and not from the real tendency to multiscaling. It should also be remembered that this type of random walk may induce some correlations because nodes with a higher multiplicity are visited more often.

Finally, an interesting global effect worth noting here is the quite visible presence of two scaling regimes of $F_{r}\left(s\right)$ in two halves of the entire *s* area considered. It may be significant that in the case of ’text-based walk’, an overall slope in the first half of this interval is smaller than in the second half, both without and with punctuation networks. This is indicated by the red dotted lines in Figure 5 generated by fitting $F_{2}\left(s\right)$, which by Equation (4) determines the corresponding Hurst exponents (H=h(2)). They are denoted as $H_{left}$ and $H_{right}$ correspondingly. Interestingly, the first ones are less than 0.5, which indicates anti-persistence, while the second ones are greater than 0.5, thus signaling persistence on larger scales. The related crossover observed corresponds to a distance of about 300 words and thus, interestingly and perhaps significantly, to about one page of traditional text. In the second case of the random walk, two regimes also stand out but with the opposite tendencies as far as the numerical values of the Hurst exponents relative to 0.5 are concerned.

**Figure 6:**
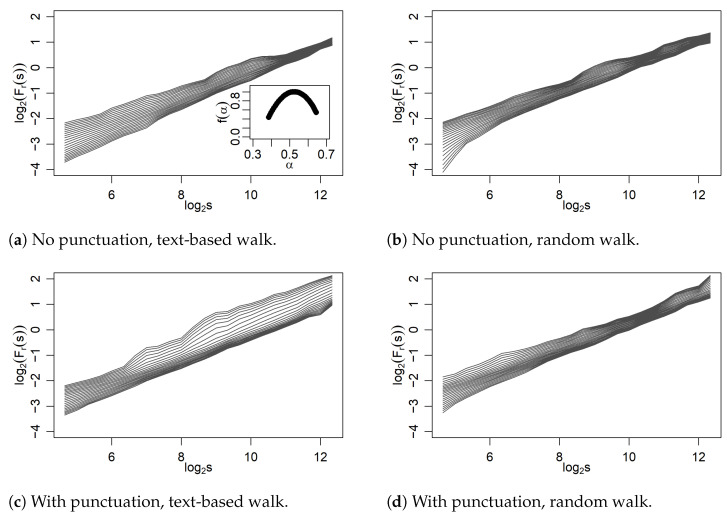
Fluctuation functions of time series constructed from the clustering coefficients of nodes visited in network walks. In (**a**), the corresponding singularity spectrum, which develops a clear multifractal scaling, is presented in the inset.

As is shown in Figure 6, the situation changes considerably if, during analogous walks on these two networks, without and with punctuation, the time series are generated by reading the appropriate clustering coefficients instead of the previous node degrees. Now, the scaling does not distinguish between two regimes and develops more in a multifractal direction. For the ‘text-based’ series on the network without punctuation, the multiscaling is even so rich that it allows us to determine the entire singularity spectrum according to Equation (5), and this spectrum is shown in the inset.

An intriguing related result shown in panel (c) of Figure 6 is that it is definitely not possible to determine an analogous spectrum for the case of a network that also takes into account punctuation. Here, the fluctuation functions $F_{r}\left(s\right)$ for different values of the parameter *r* move away from each other very significantly as the scale *s* increases. This is a very unusual picture in this type of analysis and may manifest a different role of punctuation in relation to words in text creation. The behavior of the fluctuation functions in this case may, for instance, originate from a superposition of different scaling components and thus may require an appropriate decomposition procedure. This is a very interesting and significant result worth further investigations. For a random walk counterpart of this series shown in panel (d) of Figure 6, such an effect disappears, and the fluctuation functions look similar to the case (b) of no punctuation.

A question arises as to why multifractality is observed for the clustering-coefficient time series, while it is not observed in the node-degree time series. A possible answer may lie in the fact that, in order to talk about multifractality, long-range correlations must exist [56,57]. In a word-adjacency network, the node degree describes the properties of that specific node and provides little information about the degrees of its neighbors (we neglect the disassortativity effect, which may produce short-range anticorrelations). Therefore, a walker moving on such a network does not generate long-range correlations. On the other hand, the clustering coefficient describes not only a given node but also its surroundings. In this sense, if a walker enters a region with a large (small) number of connections between nodes, it will encounter a sequence of nodes with elevated (lowered) values of the coefficient along its path, which may generate long-range correlations in time and, thus, multifractality.

### 3.3. Testing Significance of Multifractality Signals

Obtaining reliable numerical results based on the above MFDFA algorithm is a delicate matter. It is easy to overestimate and misinterpret results, especially for relatively short time series. Standard procedures for creating correlation-free surrogates by shuffling relatively short—even several thousand data points—original series may show the appearance of multifractal scaling, while true multifractality is generated only by correlations [56]. The problem of obtaining a false multifractality signal in the absence of correlations appears particularly persistently when the distributions of data fluctuations in the series are heavy-tailed. Then, very long series are needed to see the true result, indicating the absence of multifractality. In practice, insufficiently long time series, especially those with heavy tails of fluctuations, may give a false signal of multifractality [57]. The newly proposed method [58] of systematically reducing heavy fluctuation tails while maintaining correlation strength provides the most systematic method for verifying the validity of a multifractal signal. The idea is [58] to project the data by using a ranking based probability density transformation in such a way as to maintain their original order in the series but systematically change the distribution of fluctuations towards the Gaussian distribution.

*q*-Gaussians turn out to be a very practical analytical parametrization of this class of distributions. These distributions may be viewed as a generalization of the Gaussian distribution, in the same way as the Tsallis entropy $S_{q}$ generalizes the Boltzmann–Gibbs entropy *S* [59,60]. The *q*-Gaussian distribution $G_{q}$ has two parameters: the shape parameter q∈(−∞,3) and the width parameter β>0. Its PDF has the following form [59]:

(6) $$p_{q}\left(x\right)=\frac{\sqrt{\beta }}{C_{q}}e_{q}\left(-\beta x^{2}\right),$$

where $e_{q}\left(x\right)$ is the *q*-exponential function defined by

(7) $$e_{q}\left(x\right)=\left\{\begin{array}{cc} {\left(1+\left(1-q\right)x\right)}^{1/(1-q)} & \mathrm{if}\;q\neq 1\;\mathrm{and}\;1+(1-q)x>0\\ 0 & \mathrm{if}\;q\neq 1\;\mathrm{and}\;1+(1-q)x\leq 0\\ e^{x} & \mathrm{if}\;q=1 \end{array}\right.$$

and $C_{q}=\int_{-\infty }^{\infty }e_{q}\left(x^{2}\right)dx$ is a normalization factor. The preservation of clear multifractality characteristics in the limit q=1, i.e., for the Gaussian distribution, is then a convincing signal that we are dealing with true multifractality generated by correlations. It may be recalled here that heavy tails influence the spread of mutlifractality but only when correlations are present. Otherwise, only monofractality may apply or, in extreme situations, bifractality, when the fluctuations are stable in the Lévy region [56].

**Figure 7:**
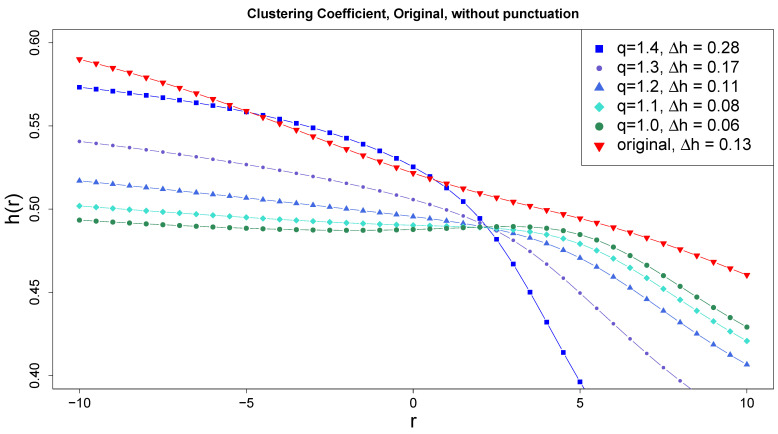
Generalized Hurst exponents for the original case of Figure 6a and for its *q*-Gaussian projected variants.

**Figure 8:**
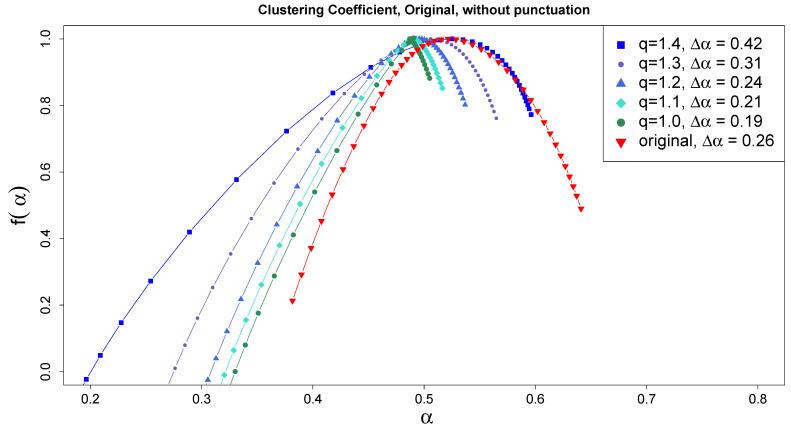
Singularity spectra corresponding to the generalized Hurst exponents of Figure 7. The same convention of symbols is used.

The case of the promisingly developed multifractality of Figure 6a is now subjected to the above test to check to what extent it is a true multifractality resulting from correlations, i.e., whether it survives the reduction in the fluctuation distribution to the Gaussian distribution. The sequence of generalized Hurst exponent h(r) for successive projections on *q*-Gaussians of the original series onto correlation-preserving series is shown in Figure 7. Clearly, even at the q=1 limit, thus for the Gaussian distribution, h(r) still depends on *r*, which means that multifractality applies. In terms of the singularity spectra, the corresponding progression and dependence are illustrated in Figure 8. Applying the same procedure to the shuffled original series, i.e., after destroying the correlations, leads to the disappearance of the manifestations of multifractality.

## 4. Summary

This study proposed and explored the application of multifractal time series analysis to linguistic networks, particularly word-adjacency networks derived from Alice’s Adventures in Wonderland by Lewis Carroll. By mapping linguistic structures onto time series and applying multifractal detrended fluctuation analysis (MFDFA), complex scaling behaviors that provide insights into the structural organization of language are uncovered. Notably, the clustering coefficient-based time series exhibited rich multifractal properties when traversed according to the reading order, suggesting an inherent complexity in natural text organization. Furthermore, the analysis confirmed the robustness of multifractal signatures through rigorous significance testing, ruling out the possibility of spurious multifractality arising from heavy-tailed fluctuations.

These findings also demonstrate that punctuation marks play a significant but distinct role in shaping the statistical properties of linguistic networks. Their inclusion alters the scaling characteristics of time series as derived from word-adjacency networks. This is particularly intriguing in light of the fact that punctuation marks obey Zipf’s law just like words [33].

The results presented here thus reveal a new perspective for quantitative linguistics and network science by providing a novel approach to studying text complexity through time series transformations. Future research can extend this methodology to diverse textual datasets, languages, and genres to explore whether these patterns are universal features of written communication. Additionally, refining network traversal techniques and integrating deeper semantic layers could further enhance our understanding of linguistic constructs. This work thus underscores the potential of interdisciplinary approaches that merge network theory, time series analysis, and linguistic studies, offering a promising framework for unraveling the intricate organization of language.

## Data Availability

These data are publicly available.
